# Anti-Inflammatory Effects of Shenfu Injection against Acute Lung Injury through Inhibiting HMGB1-NF-*κ*B Pathway in a Rat Model of Endotoxin Shock

**DOI:** 10.1155/2019/9857683

**Published:** 2019-11-03

**Authors:** Xia Liu, Fei Ai, Hui Li, Qin Xu, Liyan Mei, Jifei Miao, Quan Wen, Chaoying Zhang, Saixia Zhang, Jianhong Zhou, Xiangyun Chen, Chunwei Chu, Junfeng Guo

**Affiliations:** ^1^School of Basic Medical Sciences, Guizhou University of Chinese Medicine, Dongqingnan Road, Guizhou Higher Education Mega Center, Huaxi District, Guiyang, Guizhou 550025, China; ^2^Second Clinical Medical College, Guizhou University of Chinese Medicine, Dongqingnan Road, Guizhou University Town, Huaxi District, Guiyang, Guizhou 550025, China; ^3^School of Basic Medical Sciences, Guangzhou University of Chinese Medicine, No. 232, Waihuandong Road, Guangzhou Higher Education Mega Center, Guangzhou 510006, China

## Abstract

Shenfu injection (SFI), a Chinese herbal medicine with substances extracted from *Ginseng Radix et Rhizoma Rubra* and *Aconiti Lateralis Radix Praeparata*, is widely used as an anti-inflammatory reagent to treat endotoxin shock in China. However, the mechanism of SFI in endotoxin shock remains to be illuminated. High mobility group box 1 (HMGB1), a vital inflammatory factor in the late stage of endotoxin shock, may stimulate multiple signalling cascades, including *κ*B (NF-*κ*B), a nuclear transcription factor, as well as tumour necrosis factor (TNF)-*α* and interleukin (IL)-1*β*, among others in the overexpression of downstream proinflammatory cytokines. An investigation into the effects of SFI on the inhibition of the HMGB1-NF-*κ*B pathway revealed the contribution of SFI to acute lung injury (ALI) in a rat model of endotoxin shock. To assess the anti-inflammatory activity of SFI, 5 ml/kg, 10 ml/kg, or 15 ml/kg of SFI was administered to different groups of rats following an injection of LPS, and the mean arterial pressure (MAP) at 5 h and the survival rate at 72 h were measured. 24 h after LPS injection, we observed pathological changes in the lung tissue and measured the mRNA expression, production, translocation, and secretion of HMGB1, as well as the expression of the NF-*κ*B signal pathway-related proteins inhibitor of NF-*κ*B (I*κ*B)-*α*, P50, and P65. We also evaluated the regulation of SFI on the secretion of inflammatory factors including interleukin-1 beta (IL-1*β*) and TNF-*α*. SFI effectively prevented the drop in MAP, relieved lung tissue damage, and increased the survival rate in the endotoxin shock model in dose-dependent manner. SFI inhibited the transcription, expression, translocation, and secretion of HMGB1, increased the expression of toll-like receptor (TLR4), increased the production of I*κ*B-*α*, and decreased the levels of P65, P50, and TNF-*α* in the lung tissue of endotoxin shock rats in a dose-dependent manner. Furthermore, SFI decreased the secretion of proinflammatory cytokines TNF-*α* and IL-1*β*. In summary, SFI improves the survival rate of endotoxin shock, perhaps through inhibiting the HMGB1-NF-*κ*B pathway and thus preventing cytokine storm.

## 1. Introduction

Acute lung injury (ALI), described by increased capillary permeability and acute inflammatory disorder, is mainly caused by endotoxemia and is common worldwide [1]. Acute respiratory distress syndrome (ARDS), a more severe form of ALI, is a leading cause of mortality with endotoxin shock [2]. Overactivation of the immune system in ALI/ARDS leads to a cascade of proinflammatory mediators, which is likely responsible for the high mortality; thus, anti-inflammatory therapy is particularly important [3].

High-mobility group box 1 (HMGB1) proteins, highly conserved proteins identified more than 30 years ago, could be secreted from the cell into the extracellular matrix following stimulation by inflammatory factors such as lipopolysaccharide (LPS) [4]. Extracellular HMGB1 has been confirmed as a damage-associated molecular pattern (DAMP), which plays a part in the development of many clinical diseases, such as ALI/ARDS, sepsis, asthma, and cancer [5–11]. Through binding to receptor for advanced glycation end products (RAGE) and toll-like receptor 2/4 (TLR-2/4), extracellular HMGB1 may activate inflammation-related signalling pathways, including NF-*κ*B and mitogen-activated protein kinase (MAPK) pathways, succeeded by the overproduction of the downstream proinflammatory mediators, TNF-*α*, IL-1*β*, and IL-8 as well [10]. Recent research findings indicated that inhibiting the HMGB1-mediated TLR4/NF-*κ*B signalling pathway facilitated the treatment of LPS-induced ALI/ARDS [12].

Shenfu injection (SFI), a kind of traditional Chinese herbal medicine, has obtained approval from the Chinese State Food and Drug Administration (medicine manufacturing approval number: Z51020664). Composed of Radix Aconiti Lateralis Preparata (*Aconitum carmichaelii* Debx.) and Radix Ginseng (*Panax ginseng* C.A. Mey.), SFI has been used for treating septic shock in China [13]. Clinical trials confirmed that SFI worked more effectively than this traditional therapy with respect to enhancing mean arterial pressure (MAP), stabilizing heart rate, dredging serum lactate, and lowering mortality in patients with septic shock [14, 15].

Experiments *in vivo* have proven that SFI inhibited endotoxin-induced pulmonary inflammation by suppressing the NF-*κ*B pathway [16, 17]. Nevertheless, it remains undecided whether SFI can attenuate ALI by inhibiting HMGB1-mediated pathways in rats with ALI induced by LPS. In summary, the potential medical functions of SFI on the expression, transportation, and secretion of HMGB1 in the lung tissues of rats with endotoxin shock was investigated here.

## 2. Materials and Methods

### 2.1. Chemicals and Reagents

Shenfu injection (SFI), containing 0.1 mg aconitine and 0.5 mg ginsenoside per millilitre, came from Ya'an Sanjiu Pharmaceutical Co., Ltd (No. 51020664) (Sichuan, China). LPS (No. L2880) and dexamethasone (DXM) (No. D1756) were obtained from Sigma-Aldrich (Saint Louis, MO, USA).

Anti-rabbit HMGB1 (No. ab79823), anti-rabbit TLR4 (No. ab13556), anti-rabbit I*κ*B-*α* (No. ab32518), anti-rabbit NF-*κ*B p65 (No. ab16502), anti-rabbit TNF-*α* (No. ab6671), anti-rabbit H3 (No. ab18521), and anti-rabbit GAPDH (No. ab128915) were purchased from Abcam (Cambridge, MA, USA). TNF-*α* Rat ELISA Kit (No. BMS622), IL-1*β* Rat ELISA Kit (No. BMS630), and HMGB1 Rat ELISA Kit were purchased from Invitrogen (Thermo Fisher Scientific, Runcorn, Cheshire, UK).

### 2.2. Animals

8-10-week-old male Sprague-Dawley rats (∼220 g body weight) were provided by the Experimental Animal Centre of Guangzhou University of Chinese Medicine (license number: scxk (Cantonese) 20130020). These rats were maintained in standard germ-free housing at 22°C and 55% humidity for seven days before the experiments. All rats had access to water and were fed standard chow *ad libitum*. The experiments followed the procedures in accordance with China's legislation on the National Institutes of Health Guidelines for the Care and Use of Laboratory Animals and gained approval from the Ethics Committee of Experimental Animals at Guangzhou University of Chinese Medicine (approval number: 201700188).

### 2.3. Experimental Protocol for ALI Model

#### 2.3.1. Observation of Blood Pressure and Mortality

The randomized grouping method was used in dividing the rats into six groups (*n* = 12): sham group, LPS group, LPS + DXM (1 mg/kg) group, LPS + SFI (5 ml/kg), LPS + SFI (10 ml/kg) group, and LPS + SFI (15 ml/kg) group. Rats were first anaesthetized via intraperitoneal injection of sodium pentobarbital (30 mg/kg), maintained by supplementary injections when necessary. The right carotid artery of the rats was exposed and cannulated to connect a pressure transducer (BL-420 Apparatus) for injections and blood pressure monitoring. LPS was dissolved in sterile water and administered (15 mg/kg) to make the rats enter a state of shock; the sham group was given saline instead. Receiving LPS and entering the shock state (a drop of ∼30% in blood pressure, approximately 0.5 h after LPS injection), the rats were treated according to the groupings above. Rats in the sham and LPS groups were given saline without the drug. Blood pressure was observed every 0.5 h for 5 h to determine the MAP. Survival study required records at 6 h, 12 h, 24 h, 48 h, and 72 h time points in order to appraise the effect of SFI.

#### 2.3.2. Serum and Lung Tissue Extraction

Animal group distribution, anaesthesia, and administration were the same as described above (*n* = 6 per group). 24 h after LPS stimulation, rats were sacrificed. Blood samples were first collected, followed by serum isolation for subsequent ELISA (saved at −80°C); tissues in the right lung were rapidly excised for PCR and western blotting (saved at −80°C) and the left for histological processing after being fixed in paraformaldehyde.

### 2.4. Pulmonary Histopathology

The pulmonary samples were obtained after being fixed 48 h in paraformaldehyde solution, embedded with paraffin according to the standard protocol, sectioned (4 *μ*m in thickness), blemished with haematoxylin-eosin (H&E), and checked for any pathological changes with a light microscope (Olympus, Tokyo, Japan) in a blinded manner. A previously described scoring system was adopted to check the pathological changes in light of hyperaemia, oedema in the alveolar wall, haemorrhage, and inflammatory cell infiltration [18].

### 2.5. ELISA

HMGB1, IL-1*β*, and TNF-*α* ELISA kits were employed in accordance with the protocols provided by manufacturers. Absorbance was determined at 450 nm, and the concentrations of cytokines in the serum were calculated according to the standard curve.

### 2.6. Western Blot Analyses

Western blotting was practiced as instructed previously [12]. Concisely, same amounts of lung tissue (50 mg) were processed for total protein extraction and for nuclear and cytoplasmic extraction, as required by the NE-PER nuclear and cytoplasmic extraction kits (Pierce Biotechnology, Rockford, IL, USA). Proteins were separated with sodium dodecyl sulphate polyacrylamide gel electrophoresis (SDS-PAGE, 10%) before being transferred into a polyvinylidene fluoride membrane. Subsequently, membranes were blocked by BSA (5%) and incubated by the aforementioned primary antibody, followed by an HRP-conjugated second antibody (Cambridge, MA, USA). Finally, chemiluminescence detection was performed using the Western Chemiluminescent HRP Substrate (Millipore Corporation, Billerica, MA, USA). Relative band intensities for each protein were normalized to GAPDH.

### 2.7. Reverse Transcription-Quantitative (RT-q) PCR

From lung tissues was total RNA extracted with a TRIzol kit (Thermo Fisher Scientific, Runcorn, Cheshire, UK) in accordance with the manufacturer's protocol. Total RNA products were then transcribed to be cDNA with oligo (dT) primers (Takara Biotechnology, Dalian, China) (Table 1). Gene expression was performed using the following procedure: pre-denaturation (95°C, 10 min) and amplification (95°C, 10 s; 60°C, 30 s; 72°C, 15 s) for forty cycles. The fold change in the target gene expression was regularized to the control gene GAPDH using 2^−ΔΔCt^ method [19].

### 2.8. Statistical Analyses

Experiments were performed three times, respectively, and in triplicate, Data were expressed as mean values ± standard deviation, and the analyses were conducted with GraphPad Prism 5.0 software (GraphPad, San Diego, CA, USA). Tukey's multiple comparison test and one-way ANOVA were used in assessing comparisons between groups. Kaplan–Meier survival analysis was used to find out survival rates, and *P* < 0.05 was considered to be statistically significant.

## 3. Results

### 3.1. SFI Treatment Improves Survival Rate and MAP of Endotoxin Shock Rats

**Figure 1:**
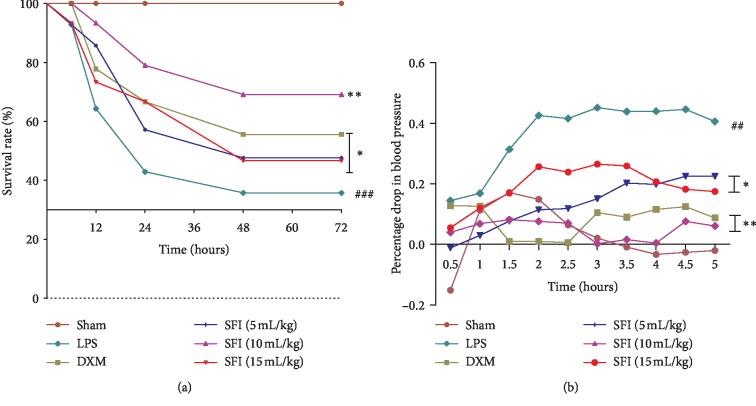
SFI improves survival and MAP in rats with endotoxin shock (*n* = 12). (a) Survival curves of different groups at the 6, 12, 24, 48, and 72 h time points. (b) MAPs of different groups were recorded every 30 min for 5 h. ^##^*P* < 0.01 and ^###^*P* < 0.001 vs. sham group; ^*∗*^*P* < 0.05 and ^*∗∗*^*P* < 0.01 vs LPS group.

To determine the therapeutic effects of SFI, the survival rate of each group was calculated (Figure 1(a)). The survival curves of the SFI groups were visibly separated: the 72 h survival rate of the LPS group was only 35.7%. In comparison to the LPS group, survival rates in different treatment groups were improved; the survival rate of the SFI 10 mL/kg group reached 71.4%, indicating that SFI had a strong protective effect on endotoxic shock rats in this experiment.

To estimate the effectiveness of SFI on MAP of the experimental rats, MAP changes were recorded every 0.5 h for 5 h (Figure 1(b)). The decline in MAP in LPS group was more than 30%, suggesting that the rats were in a state of persistent shock. By contrast, the treatment groups effectively elevated the MAP of endotoxic shock rats and facilitated recovery from shock. Moreover, 10 mL/kg SFI evidently reversed the MAP drop for shock rats (*P* < 0.01), an effect similar to that in the DXM group.

### 3.2. SFI Attenuates ALI in Endotoxin Shock Rats

**Figure 2:**
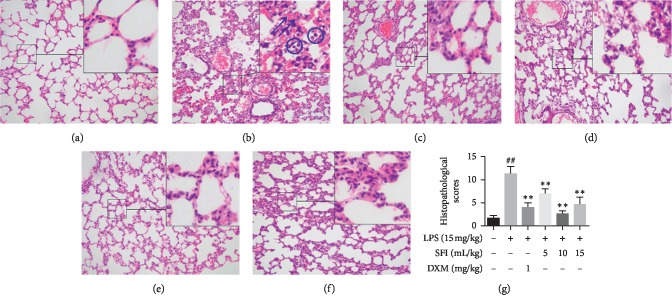
SFI attenuates the pathological impairment of lung tissue in rats with endotoxin shock. H&E staining; magnification, ×200 (*n* = 6). (a) Lung tissues of rats in the sham group under a light microscope; (b) lung tissues of rats in the LPS group showed severe acute lung injury; arrows (⟶) indicate pulmonary capillary hyperaemia and circles (○) indicate inflammatory cell infiltration. (c) Lung tissues from rats in the DXM group; (d) lung tissues from rats in the 5 mL/kg SFI group; (e) lung tissues from rats in the 10 mL/kg SFI group; (f) lung tissues from rats in the 15 mL/kg SFI group; (g) histopathological scores of lung tissues in rats of different groups. ^##^*P* < 0.01 vs. sham group; ^*∗∗*^*P* < 0.01 vs. LPS group.

To observe the effects of SFI on the pathological impairment of lung tissue, H&E staining was conducted (Figure 2). Limited histological changes were present in the lung tissues of the sham group (Figure 2(a)). However, alveolar wall hyperaemia, interstitial oedema, and notable inflammatory cell infiltration appeared in the lungs of rats belonging to the LPS group, suggesting a typical pathological inflammatory response (Figure 2(b)). Morphological observation showed that DXM and SFI (5, 10, and 15 mL/kg) treatments notably attenuated the severity of pulmonary lesions (Figures 2(c)–2(f)). Comparatively, the effects of SFI and DXM were better than those of other treatment groups. As shown in Figure 2(g), the lung tissue histopathology scores were strikingly amplified in the LPS group but were notably lessened following treatment with SFI and DXM.

### 3.3. SFI Inhibits the mRNA Expression of HMGB1-NF-*κ*B Pathway-Related Proteins and Inflammatory Factors in the Endotoxin Shock Rats' Lung Tissues

**Figure 3:**
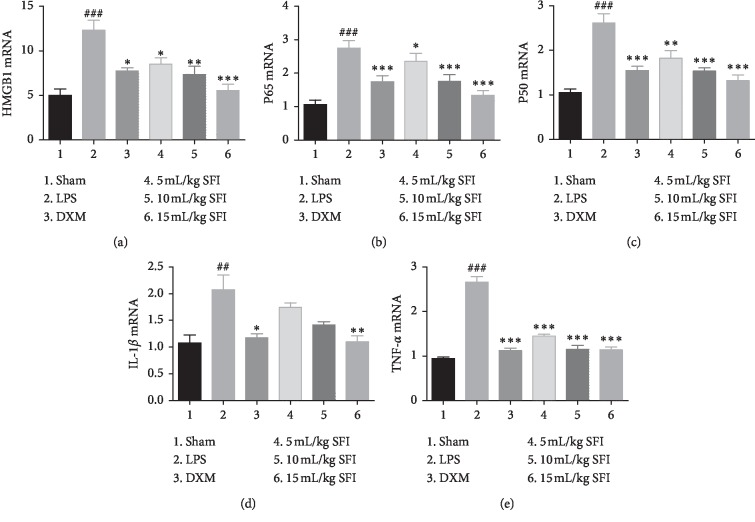
SFI inhibits the mRNA of HMGB1-NF-*κ*B pathway-related proteins and inflammatory factors in lung tissues in rats with endotoxin shock (*n* = 6). (a) HMGB1, (b) P65 mRNA, (c) P50 mRNA, (d) IL-1*β* mRNA, and (e) TNF-*α* mRNA in lung tissues of rats with endotoxin shock. ^##^*P* < 0.01 and ^###^*P* < 0.001 vs. sham group; ^*∗*^*P* < 0.05, ^*∗∗*^*P* < 0.01, and ^*∗∗∗*^*P* < 0.001 vs. LPS group.

To appraise the effects of SFI on the transcription of HMGB1-mediated NF-*κ*B signalling pathway-related proteins and inflammatory factors in the lung tissues of endotoxic shock rats, the mRNA levels of HMGB1, P65, P50, Il-1*β*, and TNF-*α* were distinguished using RT-qPCR (Figure 3). Different from the sham group, the transcription of HMGB1, P65, and P50, and proinflammatory factors TNF-*α* and Il-1*β*, in the lung tissues of the LPS group were increased significantly, while DXM and SFI treatment inhibited the transcription of these genes in a dose-dependent manner. The effects of 10 mL/kg and 15 mL/kg SFI were similar to those of DXM.

### 3.4. SFI Inhibits the Expression of HMGB1-NF-*κ*B Pathway-Related Proteins and Inflammatory Factors in the Lung Tissues of Endotoxin Shock Rats

**Figure 4:**
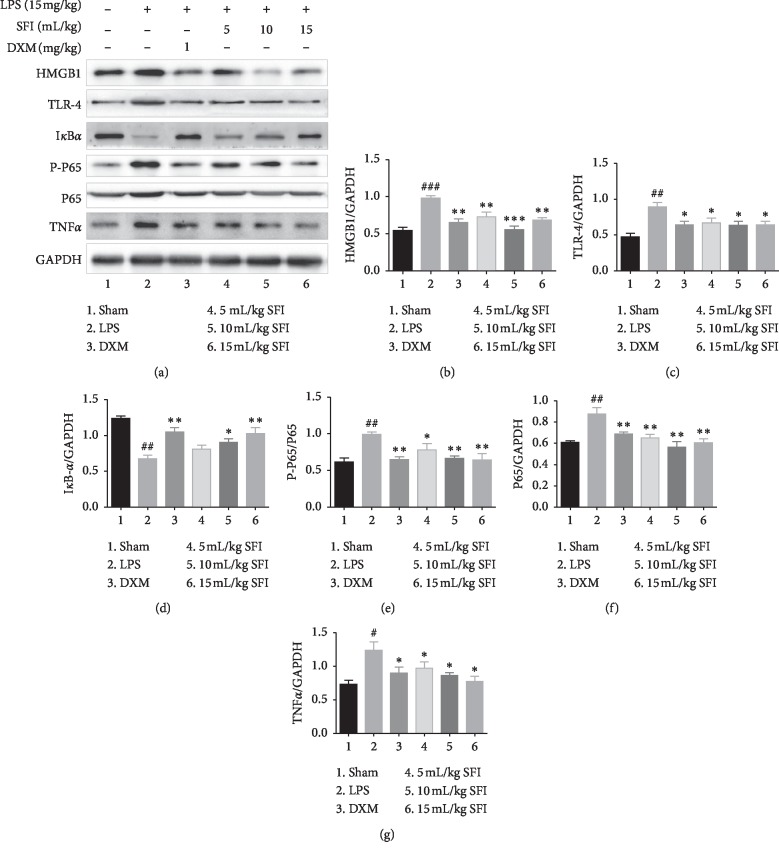
SFI inhibits the expression of HMGB1-NF-*κ*B pathway-related proteins in lung tissues of rats with endotoxin shock (*n* = 6). (a) HMGB1-NF-*κ*B pathway-related protein expression, as determined by western blotting. (b) HMGB1, (c) ILR-4, (d) I*κ*-B*α*, (e) P-P65, (f) P65, and (g) TNF-*α* levels in lung tissues of rats with endotoxin shock. ^#^*P* < 0.05, ^##^*P* < 0.01, and ^###^*P* < 0.001 vs. sham group; ^*∗*^*P* < 0.05, ^*∗∗*^*P* < 0.01, and ^*∗∗∗*^*P* < 0.001 vs. LPS group.

To investigate the potential mechanisms, protein expression in the HMGB1-mediated NF-*κ*B pathway was identified. We examined HMGB1, ILR-4, I*κ*-B*α*, P-P65, P65, and TNF-*α* in the lung tissues of rats with endotoxin shock (Figure 4). The expression of HMGB1, ILR-4, P-P65, and p65 was upregulated, and the production of I*κ*-B*α* was downregulated in the LPS group, suggesting that the HMGB1-NF-*κ*B pathway was activated. However, exposure to DXM and SFI (5, 10, and 15 mL/kg) inhibited this activation. 10 mL/kg and 15 mL/kg SFI had similar effects, and both were better than 5 mL/kg SFI. Moreover, SFI also inhibited the overexpression of TNF-*α* induced by LPS.

### 3.5. SFI Inhibits the Translocation of HMGB1 and P65 in the Nuclei and Cytoplasm of Lung Tissue Cells in Rats with Endotoxin Shock

**Figure 5:**
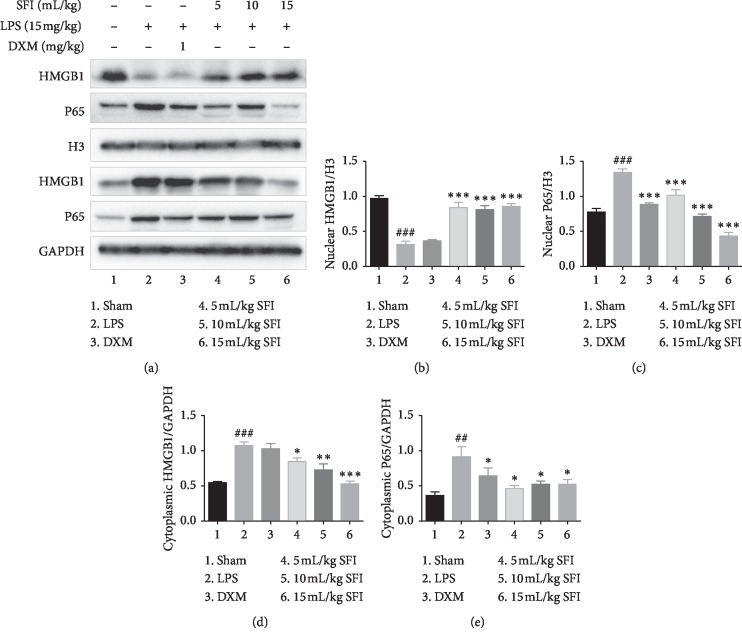
SFI inhibits the translocation of HMGB1 and P65 between the nuclei and cytoplasm in lung tissue cells in rats with endotoxin shock (*n* = 6). (a) The bands of HMGB1 and P65 proteins in the nuclei and cytoplasm were determined separately by western blotting. (b) Nuclear HMGB1, (c) nuclear P65, (d) cytoplasmic HMGB1, and (e) cytoplasmic P65 levels in lung tissues of rats with endotoxin shock. ^##^*P* < 0.01 and ^###^*P* < 0.001 vs. sham group; ^*∗*^*P* < 0.05, ^*∗∗*^*P* < 0.01, and ^*∗∗∗*^*P* < 0.001 vs. LPS group.

Translocation of HMGB1 and P65 facilitated the effective activation of the HMGB1-NF-*κ*B signalling pathway, so we observed the process in this experiment. As shown in Figure 5, cytoplasmic HMGB1 amplified sharply while nuclear HMGB1 decreased noticeably in the LPS group. SFI can inhibit HMGB1 in the cytoplasm and increase HMGB1 protein in the nucleus in a dose-dependent manner, signifying that SFI can inhibit HMGB1 translocation from the nucleus to cytoplasm. In contrast, DXM treatment did not significantly inhibit the extranuclear migration of HMGB1.

Regarding the P65 protein, both the cytoplasmic and nuclear protein were upregulated meaningfully, revealing that the previously inactive NF-*κ*B pathway became activated in the LPS group. DXM and SFI (in a dose-dependent manner) significantly depressed the production of nuclear and cytoplasmic P65 protein.

### 3.6. SFI Inhibits the Secretion of Proinflammatory Factors in the Plasma of Rats with Endotoxin Shock

**Figure 6:**
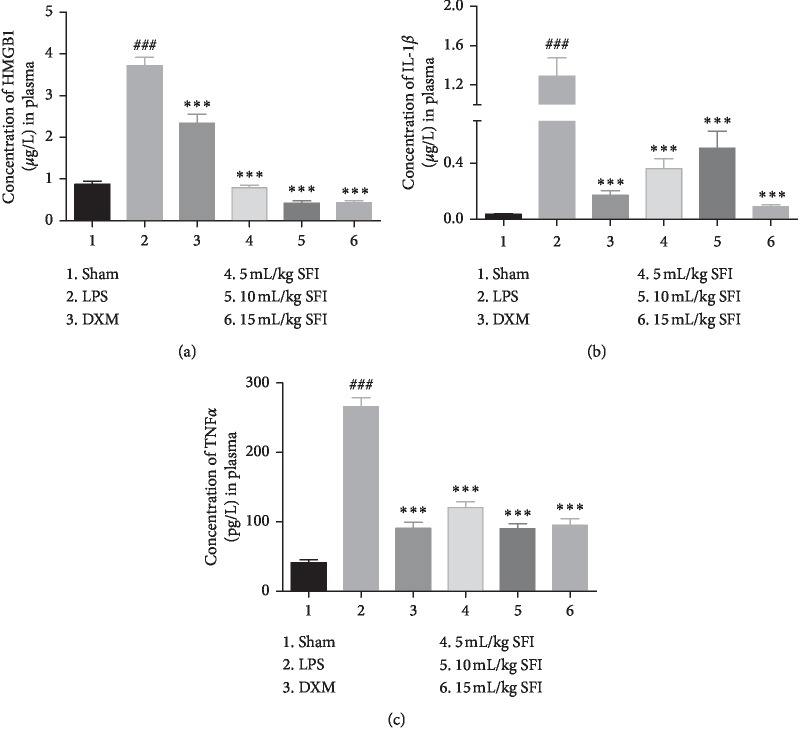
SFI inhibits the secretion of proinflammatory factors in the plasma of rats with endotoxin shock (*n* = 6). Bar graphs of the concentrations of (a) HMGB1, (b) IL-1*β*, and (c) TNF-*α* in the plasma of rats with endotoxin shock. ^###^*P* < 0.001 vs. sham group; ^*∗∗∗*^*P* < 0.001 vs. LPS group.

ELISA kits were used to quantify the serum concentrations and detected a remarkable release of HMGB1, TNF-*α*, and IL-1*β* in ALI rats with endotoxin shock (Figure 6). The secretion of these inflammatory factors was found to be inhibited by DXM and SFI treatment (in a dose-dependent manner).

## 4. Discussion

ALI is frequently complicated with endotoxin shock; investigations have shown that about 50% of endotoxin shock patients have accompanying ALI and excessive inflammation in lung tissue [1, 2]. Considering that the excessive release of inflammatory factors is one of the key pathological reactions in endotoxin shock ALI, inhibiting the cascade amplification of inflammation is an important approach to treatment. The present study was targeted to assess the anti-inflammation effect of SFI on the HMGB1-NF-*κ*B signalling pathway *in vivo*. The findings demonstrated that SFI improved the reduction in MAP, facilitating the recovery of rats after the shock. Survival rate is a key factor in evaluating the efficacy of SFI; this increased from 35.7% in the LPS group to 71.4% in the 10 mL/kg SFI group. Moreover, SFI alleviates pathological changes, such as pulmonary oedema, haemorrhage, and inflammatory cell infiltration, in the tissue. These results support SFI as a valuable therapeutic agent adopted for the treatment of ALI/ARDS. Interestingly, the effect of 10 mL/kg SFI was superior to 15 mL/kg SFI; thus, we speculate that 10 mL/kg SFI might be the optimal dose in this experiment. Nevertheless, the mechanism triggering the effect of SFI on ALI is still unclear.

HMGB1 was initially considered to be a conservative, bounteous, and permeating chromatin-related protein, essential for survival [20, 21]. However, hyperacetylation of HMGB1 can alter its subcellular localization, transferring a predominantly nuclear to a cytoplasmic location and subsequent secretion to the extracellular matrix [4, 22]. Previous experiments have confirmed that extracellular HMGB1 is a lethal inflammatory mediator in late sepsis as it activates the inflammation-related signalling pathways by binding with its specific receptors RAGEs/TLR-2/TLR-4. [7, 23–26]. The HMGB1-TLR4/NF-*κ*B signalling pathway found its way in the development of ALI/ARDS; inhibiting the overexpression and release of HMGB1 is believed to be an effective approach to ALI/ARDS treatment [6, 12].

SFI, a traditional Chinese medicine, has been widely used to treat infectious shock and haemorrhagic shock in China and has been proven to have an effect in septic shock patients [13–15]. Experiments have confirmed the pharmacological effects of SFI, including scavenging free radicals [27], suppressing cell apoptosis [28], decreasing the proinflammatory mediators [29], regulating the host immune response, and improving cellular immunity [15]. The main active constituents in SFI are ginsenosides and aconitum alkaloids, and there have been many studies confirming the pharmacological effects of ginsenoside Rg1, ginsenoside Rb1, ginsenoside Re, and hypaconitine [30]. Ginsenoside Rg1 has been demonstrated to be able to restrain inflammatory responses via downregulating NF-*κ*B activity, depressing caspase 3 activation, and inhibiting pulmonary cell apoptosis [17, 31]. Previous studies also showed that ginsenoside Rb1 also ameliorated the lung injuries by downregulating the NF-*κ*B pathway [32]. Ginsenoside Re could reverse methamphetamine-induced oxidative burdens, mitochondrial dysfunction, proinflammatory changes, apoptosis, and dopaminergic degeneration via inactivating protein kinase C*δ* [33]. Hypaconitine could suppress the apoptosis of endothelial cells by increasing the degree of deacetylation of HMGB1 in an oxidized low-density lipoprotein cell model [34]. In conclusion, the active ingredients of Shenfu injection can inhibit inflammation injury through different ways.

At present, most experiments about SFI inhibiting inflammatory factors focus on early inflammatory factors, including TNF-*α*, IL-1*β*, and IL-6; however, the treatment time window for early inflammation is relatively limited [17, 29]. HMGB1, serving as a late mediator of endotoxin lethality, is released into the serum later than the early proinflammation factors; its peak level was at 16–24 h and lasted for ∼72 h after stimulation with proinflammatory factors [7, 35]; therefore, this experiment focussed on HMGB1, the late inflammatory factor. This study discovered a significant increase of HMGB1 in pulmonary tissues, which was accompanied by increased cytoplasmic HMGB1 and decreased nuclear HMGB1, indicating the transfer of the subcellular localization of HMGB1 from the nucleus to the cytoplasm in endotoxin shock rats. Meanwhile, HMGB1 secreted to the extracellular matrix, thus, plasma HMGB1 levels increased significantly when stimulated by LPS. SFI suppressed HMGB1 secretion and cytoplasmic HMGB1 and brought the nuclear HMGB1 back to the previous level. The results provide strong evidence that the HMGB1-related signalling pathway functions in the inflammatory cascade in LPS-induced shock ALI and that SFI might serve as a valued HMGB1-targeting drug due to its ability to inhibit the transcription, expression, translocation, and secretion of HMGB1 in the lung tissue of rats with LPS-induced endotoxin shock.

The production of inflammatory factors mainly depends on the effective activation regarding the NF-*κ*B signalling pathway. Members of the NF-*κ*B family exist in the form of homologues or heterodimers; a furthermost common form is the P65-P50 dimer that is usually located in the cytoplasm in an inactive state and is bound to I*κ*-B*α*, its inhibitor. Conversely, in answer to proinflammatory factors, I*κ*-B*α* protein is firstly degraded by ubiquitination, followed by the free P65-P50 dimer migrating into the nucleus and binding to the *κ*B site, thus initiating the transcription progress of inflammatory factors such as TNF-*α* and IL-1*β* [36, 37]. NF-*κ*B-mediated inflammation stays a critical mechanism in endotoxin shock. A previous study proved that SFI tended to play a key role in battling inflammation by inhibiting the NF-*κ*B signalling pathway; this may occur through its active components: diester-type alkaloids, protopanaxadiol glycosides, protopanaxatriol glycosides, and aconine derivatives [38]. Our study found that SFI blocked the NF-*κ*B signalling pathway by upregulating I*κ*-B*α* protein, downregulating phosphorylated P65, and inhibiting the expression of P65 (nuclear and cytoplasmic) in the lung tissues of ALI rats with endotoxin shock. This is an important contributor to SFI attenuating ALI in endotoxic shock rats.

By detecting the expression, subcellular localization, and secretion of HMGB1; the production of TLR-4; the expression of NF‐*κ*B signalling pathway-related proteins; and the intervention of SFI on these proteins in the lung tissues of endotoxin shock rats, we conclude that the effect of SFI on ALI induced by endotoxin shock at least partially depends on the inhibition of the HMGB1/TLR4/NF‐*κ*B signalling pathway.

To sum up, SFI's anti-inflammatory effects come from suppressing the expression, translocation, and secretion of HMGB1, thus suppressing the HMGB1-mediated NF-*κ*B signalling pathway and blocking the expression of downstream inflammatory factors such as TNF-*α* and IL-1*β*. Therefore, this may be listed as one important molecular mechanism specifying the effects of SFI in the treatment of endotoxic shock ALI.

## Figures and Tables

**Table 1:** Primers used for RT-qPCR.

Gene name	Forward primer	Reverse primer
HMGB1	5′-TTCTGTTCTGAGTACCGCCCA-3′	5′-TTGTCATCCGCAGCAGTGTT-3′
P65	5′-ACCTGGAGCAAGCCATTAGC-3′	5′-CGGACCGCATTCAAGTCATA-3′
P50	5′-CAACATCTCCTTGGCTGGCT-3′	5′-TCCGGCCGCTATATGCAG-3′
IL-1*β*	5′-GCAGCATCTCGACAAGAGCTT-3′	5′-GCTCCACGGGCAAGACATAG-3′
TNF*α*	5′-CCGAGTGACAAGCCTGTAG-3′	5′-CAATGATCCCAAAGTAGACCT-3′
GAPDH	5′-CCTGCACCACCAACTGCTTAG-3′	5′-TCTTCTGGGTGGCAGTGATG-3′

## Data Availability

The data used to support the findings of this study are available from the corresponding author upon request.

## Supplementary Materials

Supplementary Materials LPS induces inflammation through promoting the activation of NF-*κ*B and the secretion of HMGB1. SFI inhibits the transcription of HMGB1 and its translocation from the nucleus to the cytoplasm and extracellular induced by LPS. Besides, SFI also suppresses the nuclear import of NF-*κ*B, which inactivates NF-*κ*B signal pathway and inhibits the expression of proinflammatory factors.
