# Supplementary material for: Anti-Inflammatory Effects of Shenfu Injection against Acute Lung Injury through Inhibiting HMGB1-NF-κB Pathway in a Rat Model of Endotoxin Shock
Source: Evid Based Complement Alternat Med. 2019 Nov 3;2019:9857683. doi: 10.1155/2019/9857683 (PMC6875290; doi:10.1155/2019/9857683)
Supplement: Supplementary Materials — LPS induces inflammation through promoting the activation of NF-κB and the secretion of HMGB1. SFI inhibits the transcription of HMGB1 and its translocation from the nucleus to the cytoplasm and extracellular induced by LPS. Besides, SFI also suppresses the nuclear import of NF-κB, which inactivates NF-κB signal pathway and inhibits the expression of proinflammatory factors. [file 9857683.f1.docx]

Graphical Abstract:
